# Metrology of convex-shaped nanoparticles *via* soft classification machine learning of TEM images†

**DOI:** 10.1039/d1na00524c

**Published:** 2021-10-13

**Authors:** Haotian Wen, Xiaoxue Xu, Soshan Cheong, Shen-Chuan Lo, Jung-Hsuan Chen, Shery L. Y. Chang, Christian Dwyer

**Affiliations:** School of Materials Science and Engineering, University of New South Wales Sydney NSW 2052 Australia shery.chang@unsw.edu.au; School of Mathematical and Physical Sciences, University of Technology, Sydney Ultimo NSW 2007 Australia; Electron Microscope Unit, Mark Wainwright Analytical Centre, University of New South Wales Sydney NSW 2052 Australia; Material and Chemical Research Laboratories, Industrial Technology Research Institute Hsinchu Taiwan; Electron Imaging and Spectroscopy Tools PO Box 506 Sans Souci NSW 2219 Australia dwyer@eistools.com; Physics, School of Science, RMIT University Melbourne Victoria 3001 Australia

## Abstract

The shape of nanoparticles is a key performance parameter for many applications, ranging from nanophotonics to nanomedicines. However, the unavoidable shape variations, which occur even in precision-controlled laboratory synthesis, can significantly impact on the interpretation and reproducibility of nanoparticle performance. Here we have developed an unsupervised, soft classification machine learning method to perform metrology of convex-shaped nanoparticles from transmission electron microscopy images. Unlike the existing methods, which are based on hard classification, soft classification provides significantly greater flexibility in being able to classify both distinct shapes, as well as non-distinct shapes where hard classification fails to provide meaningful results. We demonstrate the robustness of our method on a range of nanoparticle systems, from laboratory-scale to mass-produced synthesis. Our results establish that the method can provide quantitative, accurate, and meaningful metrology of nanoparticle ensembles, even for ensembles entailing a continuum of (possibly irregular) shapes. Such information is critical for achieving particle synthesis control, and, more importantly, for gaining deeper understanding of shape-dependent nanoscale phenomena. Lastly, we also present a method, which we coin the “binary DoG”, which achieves significant progress on the challenging problem of identifying the shapes of aggregated nanoparticles.

## Abstract 2

The shape of nanoparticles is a key performance parameter for many applications, ranging from nanophotonics to nanomedicines.

## Graphical abstract

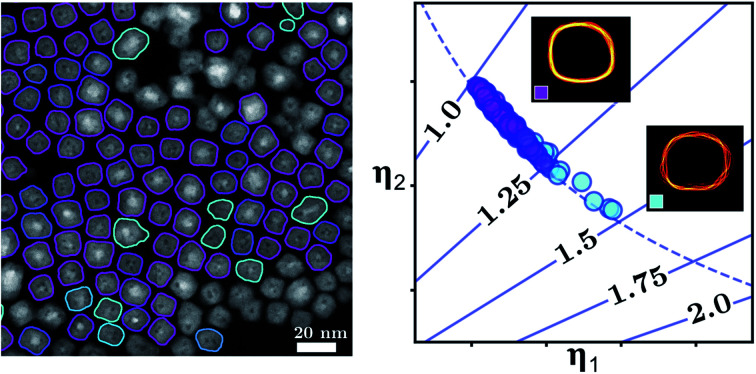

## 1. Introduction

Controlling the shape of nanoparticles is key to their successful application in many technologically-relevant fields, from the more-established disciplines of photonics,^1,2^ plasmonics,^3^ and catalysis,^4,5^ to the recently-developed field of nanomedicines.^6,7^ While new synthesis techniques now enable the generation of a huge variety of shapes,^8,9^ shape inhomogeneity is a generally recognised problem, even in precision-controlled laboratory-scale synthesis. Nanoparticles of different shapes expose different ratios of crystallographic surfaces, and therefore have different functional properties. For example, photonic nanoparticles show shape dependence in absorption properties^2,10^ as well as in emission wavelength broadening^2,11,12^ and intensity variations.^13^ Recently, the shapes of nanoparticles have been shown to have a significant effect in nanomedicine applications. The nanoparticle shape can affect the cellular uptake,^14–16^ biomolecule absorption^15^ and even their cytotoxicity.^16,17^ Considering the importance of nanoparticle shapes to their functional properties, the capacity to rapidly and accurately quantify the nanoparticle shape distributions is critical.

Precise metrology of nanoparticle shapes ultimately requires high spatial resolution. Transmission electron microscopy (TEM) is a very well-suited technique, since it can provide direct information on nanoparticle structure down to the atomic level.^18–20^ On the other hand, the number of nanoparticles that can be analyzed with TEM is traditionally far fewer than bulk techniques, such as small-angle X-ray scattering,^21,22^ since any manual analysis of TEM images of statistically representative numbers of particles is very laborious. In order to overcome this, it is desirable to utilise machine learning approaches for nanoparticle shape metrology.

Machine learning of TEM data has gained much attention in recent years. It has seen application across a broad range of materials and TEM modalities, including imaging and diffraction, for example, to resolve atomic structures of defects in 2D materials,^23^ dynamic nanoparticle structure evolution,^24^ crystal structure determination in electron diffraction,^25^ and protein and inorganic particle classification in TEM (and SEM).^26,27^ Nearly all of these previous reports have used supervised machine learning, or deep learning, methods which necessitate training data sets generated from experiments and/or simulations. However, in the case of nanoparticle shape metrology, considering the varieties of particle shapes that can be synthesized, and their unintended, uncontrolled variants, such supervised approaches are undesirable, since they require *a priori* assumptions or databases on the very particle properties which require optimization.

Here we have developed a new machine learning approach to nanoparticle metrology *via* a Hu moments-based soft classification (HuSC). The HuSC method employs Hu moments^28^ extracted from TEM image data as nanoparticle shape descriptors, and applies expectation maximization of a Gaussian mixture model^29^ to achieve soft shape classification. The use of soft classification forms an extension of our recent work.^61^ HuSC is a general, probability-based, classification method which does not require any *a priori* knowledge of the particle shapes. Moreover, unlike previous works^30–33^ which have either explicitly or effectively employed hard classification, which can be accomplished by *k*-means,^29,34^ for example, the soft classification employed in HuSC provides considerably greater flexibility in that it is applicable to both systems of nanoparticles with distinct shapes (*e.g.*, nanorods, nanoprisms, *etc.*) as well as systems with non-distinct shapes where hard classification fails to provide meaningful results. Such flexibility often becomes crucial for the analysis of nanoparticle batches produced by large-scale industrial methods, where inhomogeneities in the synthesis conditions often result in broad shape distributions including irregular, non-distinct shapes.

We demonstrate our HuSC method on three disparate nanoparticle systems. The results demonstrate that the method offers quantitative, meaningful statistical descriptions of convex particle shapes for both highly-controlled laboratory-synthesized nanoparticle systems as well as large-scale fabrications used in industry. Our presentation here is confined to systems containing convex-shaped particles, however, the ideas presented here are also applicable, with little modification, to non-convex particles.

## 2. HuSC: Hu moments soft classification method

**Fig. 1:**
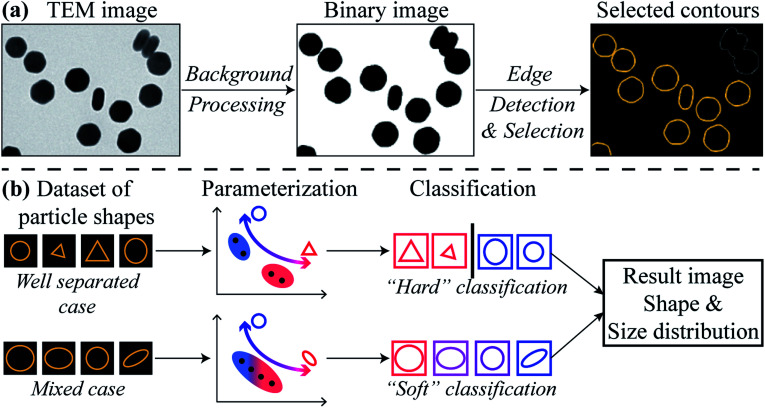
Workflow of nanoparticle shape metrology using HuSC method from TEM images. (a) Converting TEM image datasets into particle contours. (b) The soft classification adopted in HuSC reduces to a hard classification for well separated shapes.

Fig. 1 illustrates the HuSC workflow for nanoparticle shape metrology. The workflow begins with converting a 2D image dataset acquired from TEM into appropriate 1D datasets of particle contours, as shown in Fig. 1(a). TEM images can be acquired using any of the common imaging modalities in the TEM, such as bright-field (BF) TEM, or annular dark-field scanning TEM (ADF-STEM). The nanoparticles are separated from the image background by first denoising and smoothing, *e.g.*, with a Fourier filter,^35^ followed by intensity thresholding to remove the background and to produce a binary-valued image of nanoparticles. Canny edge detection^36^ is then used to extract the contour of each particle, *i.e.*, the closed contour coinciding with the edge of a particle, specifying its 2D shape. Canny edge detection is very reliable when particles are well separated. The HuSC method for shape classification and metrology is illustrated in Fig. 1(b). The method broadly consists of parameterizing the particle shapes, followed by probabilistic “soft” classification.

The study of object shapes using contours is a field with a significant history. The parameterization of a contour's shape generally involves one or more “shape descriptors”, of which several well-established formulations exist, such as Fourier descriptors,^37–39^ wavelet descriptors,^40,41^ and image moments, including Hu moments,^28,42^ complex moments,^43,44^ and Zernike and other moments.^43^ Most (if not all) of these formulations are compatible with the notion of soft classification. The choice of one formulation over another involves a compromise between shape discriminating power and interpretability.

Here, we adopt the (logarithms of the) Hu moments,^28^ which are a set of (up to seven) moments that depend on the contour's shape but are independent of its overall size, orientation and position. Hence the shape of a given nanoparticle is represented as a single point in a multidimensional “Hu space”. Such a description has been used in previous works.^45,46^ However, we have found that it is often not necessary to use the full set of Hu moments, and, moreover, that using a reduced set when it is possible can increase the reliability of the method and simplify the interpretation. Generally, more complex shapes will require a greater number of Hu moments, and/or possibly other shape descriptors, in order to distinguish them. In the examples below involving convex-shaped particles, we have used only the first two Hu moments, although we stress that our method is ultimately far more general. The first two Hu moments *H*_1,2_ are related to the two principal moments of inertia *η*_1,2_ of a filled contour of unit area, *via* the relation1
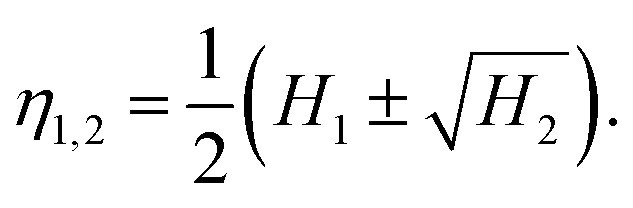


The moments *η*_1,2_ are the eigenvalues of the 2D inertia tensor. For an ellipse contour scaled to have unit area, *η*_1,2_ are the semi-major and semi-minor axes of the ellipse.

Given a set of points in Hu space which correspond to the nanoparticles in a TEM image dataset, classification of their shapes proceeds with probability-based, expectation-maximization of a Gaussian mixture model.^29^ Such mixture models achieve “soft” classification, in which a given nanoparticle can be assigned to multiple shape classes with differing weightings. The concept of soft classification is substantially better suited to nanoparticle metrology than “hard” classification schemes (*e.g.*, *k*-means) in which a given particle must belong to only one particular class. The resulting HuSC method accommodates both cases where the nanoparticle shapes are distinct and cases where they are non-distinct. In the former cases, the soft classification naturally reduces to a hard classification and, moreover, the number of classes will naturally reduce to the correct number.

In the final steps, the classified contours are further analyzed to produce particle size and shape distributions and other relevant information. Further details of the above methodology are provided in the ESI.†

## 3. Results and discussion

Here we demonstrate our HuSC method by way of three examples involving convex-shaped nanoparticles. The examples cover a range of nanoparticle shapes, varying degrees of particle dispersion, and two different TEM imaging modalities.

### 3.1 Well dispersed, distinct shaped nanoparticles

Our examples begin with a relatively ideal case, namely, well-dispersed nanoparticles synthesized in a laboratory with well-defined shapes and sizes, and which give rise to high contrast in BF-TEM imaging. The sample consists of NaGdF_4_:49%Yb,1%Tm upconversion nanoparticles (UCNPs). UCNPs capable of converting near-infrared excitation into UV and visible emissions are becoming a sought-after optical nanomaterial for use in biomedical fields including deep-tissue optical bio-imaging,^47^ multiple imaging modalities,^48^ and photothermal/dynamic treatment.^49,50^ Since the quantum yield is intrinsically low, careful control of the UCNPs morphology, including shape (and size), is critical to their photonic, electronic and magnetic properties.

**Fig. 2:**
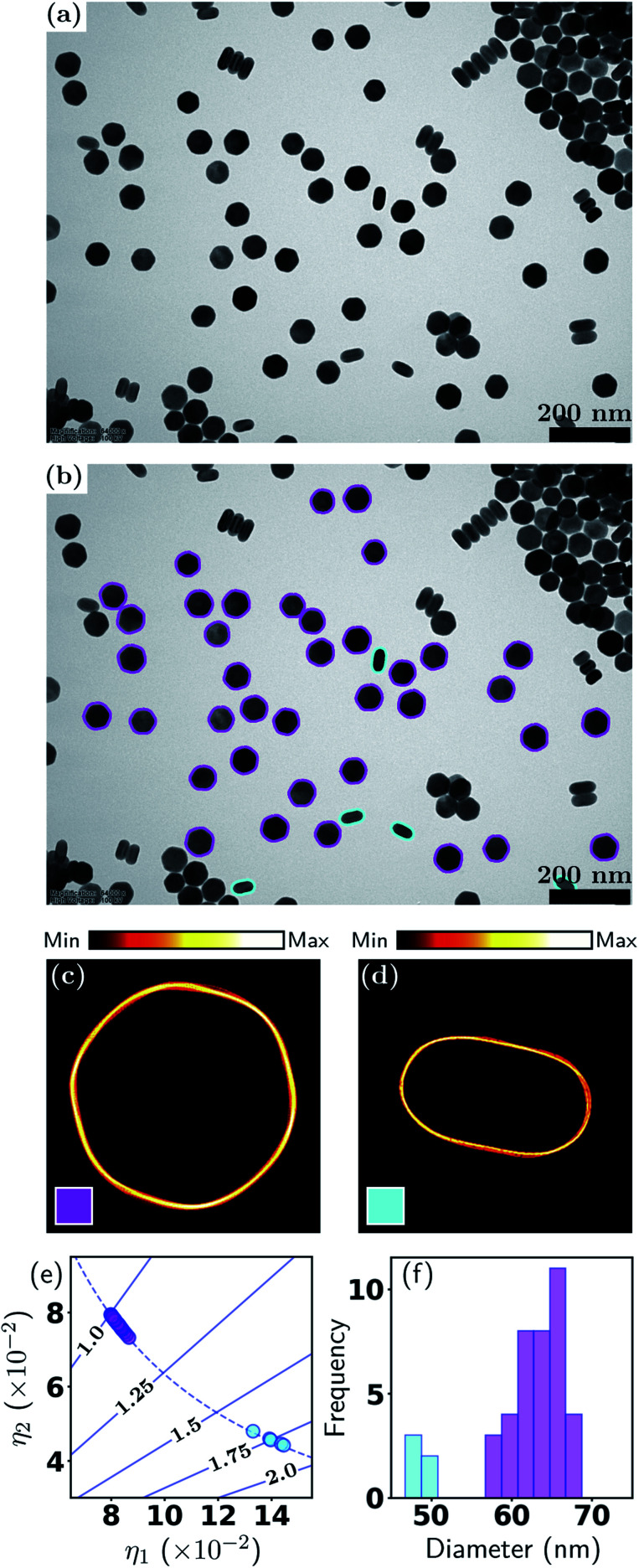
Metrology of UCNPs *via* HuSC machine learning method. (a) BF-TEM image; (b) contours of hexagonal (magenta) and rod shaped (cyan) particles overlaid on (a); (c and d) density plots of scale and orientation matched contours; (e) scatter plot of particle shape eigenvalues (solid lines indicate aspect ratios, dashed line is trend for general ellipse); (f) distribution of effective diameters.

Fig. 2 shows the results of the HuSC method applied to BF imaging of the UCNPs (Fig. 2(a)). In Fig. 2(b) we show the BF-TEM image with the contours of 43 isolated particles overlaid and color coded according to their two classifications: hexagonal (magenta) and rod-shaped (cyan). The contours of aggregated particles were discarded here. Classification of the aggregated particles is discussed in Section 3.4. In this example, the particle shapes are well defined, and the soft classification algorithm effectively produces a hard classification where the number shape classes automatically reduces to 2 (consistent with a visual inspection). Fig. 2(c) and (d) show density plots of the hexagonal and rod-shaped contour classes. A density plot is formed by overlaying the scale- and orientation-matched contours, where each contour is weighted according to the responsibility of the class. Here, where the shapes are distinct, the responsibilities are either 0 (none) or 1 (full), and the density plots are extremely tight, indicative of the well-defined shapes. In Fig. 2(c) the facets of the hexagons are clearly seen.

Key information related to the UCNP performance, namely, the particle shape and size, are plotted in Fig. 2(e) and (f). Fig. 2(e) shows the shape eigenvalues *η*_1,2_ of each particle (points), along with the aspect ratios (solid lines) and the relationship for perfect ellipses of varying aspect ratios (dashed line). Note that “size” is normalized out of this plot, so that the data points and the ellipse curve contain only shape information. One class has aspect ratios close to 1 (hexagons, magenta), and the other class has aspect ratios spread around 1.75 (rod-shaped, cyan). In this example, all particle shapes lie very close to the ellipse curve, and hence *η*_1,2_ can be interpreted as the semi-major and semi-minor axes of the particles (after size normalization). Fig. 2(f) shows the distribution of effective diameters. In this case, the diameters of the two classes are well separated. The analysis reveals that, even for well-defined hexagonal shaped particles, the size distribution has a standard deviation of about 5 nm, which can affect the distributions of Gd, Yb and Er ions on the nanoparticle surfaces, and therefore directly and significantly impact the nuclear magnetic resonance signals from Gd ions and the intensity of upconverted UV/visible light. Table 1 provides a summary of the statistics, in which *k* is the class number, ∑ and fraction refer to the effective number (total responsibility) and fraction of particles in each class, and diameter and aspect ratio (AR) refer to the class-averaged quantities (the errors represent one standard deviation). An extension of the present example, which uses multiple TEM images and more nanoparticles, is presented in Section S2, Fig. S1 and Table S1 of the ESI.†

**Table 1:** Summary for UCNPs shape analysis, where *k* denotes the shape class, ∑ and fraction are the effective number (total responsibility) and fraction of particles, diam. and AR are the particle diameter and aspect ratio

*k*	∑	Fraction	Diam. (nm)	AR
1	38	88.3%	64.5 ± 3.0	1.03 ± 0.02
2	5	11.7%	49.0 ± 1.1	1.76 ± 0.05

### 3.2 Closely packed, irregularly shaped nanoparticles

The previous example involved a relatively ideal nanoparticle system and TEM imaging conditions. However, in reality, many nanoparticle systems do not have well-defined shapes, and the TEM images often have high particle packing densities and highly fluctuating backgrounds. Such conditions can arise from the properties of the nanoparticles themselves, limited control over particle shape (and size) in the syntheses, nonideal TEM imaging conditions, or the researcher's need to increase the particle packing density to obtain larger datasets. In this example, we demonstrate our method for closely packed particles of irregular shapes, and a relatively low particle-to-background contrast. The dataset consists of commercial, industry-scale CdS/ZnSe quantum dots (QDs) imaged using BF-TEM.

**Fig. 3:**
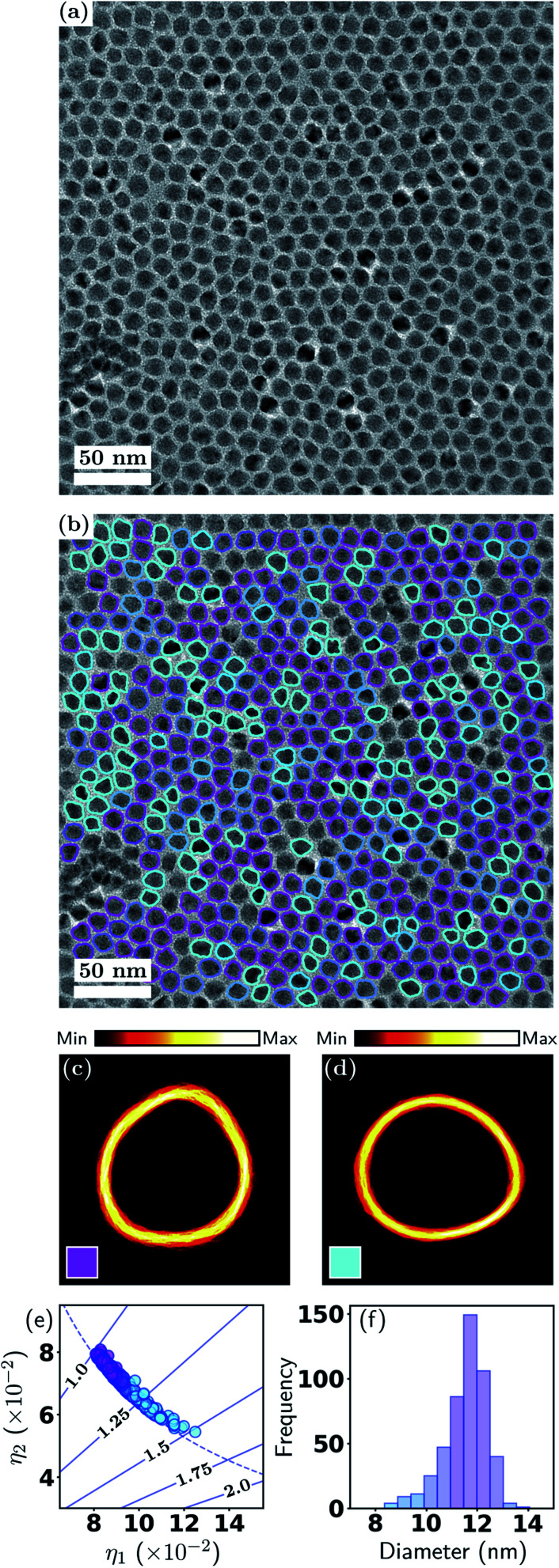
Metrology of QDs. (a) BF-TEM image and (b) with contours; (c and d) density plots of contour classes; (e) scatter plot of shape eigenvalues; (f) effective diameter distribution. See Section 3.1 and Fig. 2 for detailed explanations.

As seen in Fig. 3(a), the QDs almost entirely fill the field of view, while the image background, whose intensity differs very little from the particles, occupies only a small proportion of the image. Moreover, as the shapes of the quantum dots are irregular, it would be extremely difficult to attempt a manual shape classification. Fig. 3(b) shows the color coded contours of 482 isolated quantum dots in the image, which demonstrates the effectiveness of our particle identification method, even in this more complex scenario. Here the QDs were classified into two shape classes, however, unlike the previous example, here the particle shapes are non-distinct, hence there is a significant degree of “class mixing”. Thus the color coding of a given contour in Fig. 3(b) is a weighted mixture of magenta and cyan, representing the responsibilities of the two classes for the given contour.

The soft classification is easily appreciated from Fig. 3(e), where it is seen that the classification forms a continuum which is strongly dependent on the aspect ratio that ranges from 1–1.5. It is apparent that the total responsibilities of each class are comparable, *i.e.*, the distribution of data points among the two classes is comparable. There is some deviation of the data points from the ellipse curve, reflecting the non-elliptical shapes. In the diameter distribution in Fig. 3(f), the color coding is again indicative of the class mixing. There is a weak but discernible correlation between outlying diameters and higher aspect ratios, a fact that can be visually verified from Fig. 3(b). The density plots in Fig. 3(c) and (d) appear significantly more diffuse compared to the previous example, again indicative of the non-distinct particle shapes. A summary of the statistics is presented in Table 2.

**Table 2:** Summary for CdS/ZnSe QDs shape analysis, where *k* denotes the shape class, ∑ and fraction are the effective number (total responsibility) and fraction of particles, diam. and AR are the particle diameter and aspect ratio

*k*	∑	Fraction	Diam. (nm)	AR
1	272.7	57%	11.9 ± 0.7	1.12 ± 0.05
2	209.3	43%	11.7 ± 0.9	1.25 ± 0.08

### 3.3 Core–shell nanoparticles with irregular shape variants

This example involves ADF-STEM imaging of Fe-core/Fe_2_O_3_-shell nanocubes. This mode of imaging is often used to study nanoparticles containing higher atomic number(s) and/or compositional variations, *e.g.*, core–shell nanoparticles.^51–53^ Magnetic nanoparticles, such as those of iron, exhibit unique magnetism that can be systematically tuned by their size and shape, and as a result have been extensively researched for applications in data storage,^54^ catalysis^55^ and biomedicine.^56,57^ Having low cytotoxicity and high magnetization, iron nanoparticles with controlled magnetic properties are ideally suited for use in bio-imaging and magnetic hyperthermia.

**Fig. 4:**
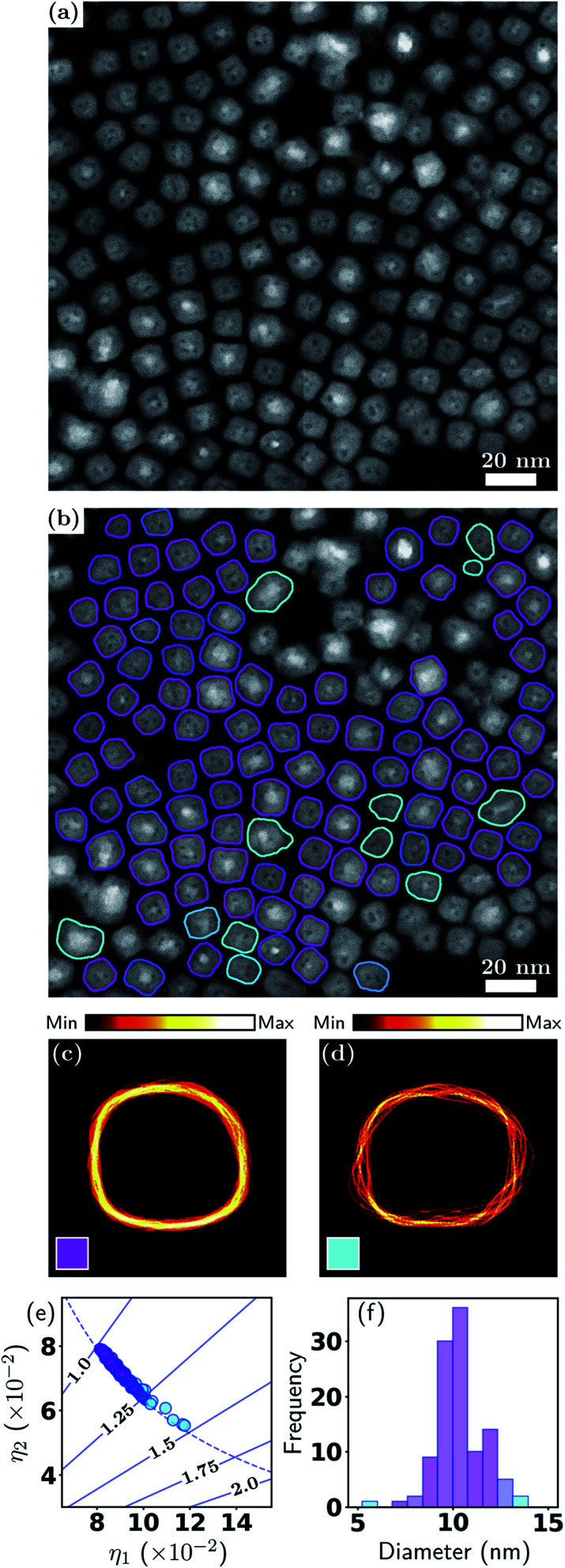
Metrology of Fe–Fe_2_O_3_ nanocubes. (a) ADF-STEM image and (b) with particle contours overlaid; (c and d) contour densities; (e) particle shape eigenvalues; (f) effective diameter distribution. See Section 3.1 and Fig. 2 for detailed explanations.

The ADF-STEM image in Fig. 4(a) exhibits a low, homogeneous background and high particle-to-background contrast. The internal structure of the particles is clearly evident, with the Fe cores giving rise to higher intensity. Fig. 4(b) shows the particle contours overlaid and color coded according to a soft classification with two shape classes. This result demonstrates the effectiveness of our method for detecting isolated particles when they exhibit internal structure, which is an important distinction from the previous example and is relevant to many nanoparticle applications.

While it is clear that the particles in Fig. 4(b) have a strong tendency for adopting cube-like shapes, it is also evident that they exhibit variations in both shape and size. This is also captured in Fig. 4(e), where it is seen that the majority of particles have aspect ratios in the range 1.0–1.25, or, stated alternatively, the two classes exhibit unequal total responsibilities, with class *k* = 1 (labelled in magenta) taking on the majority of responsibility. Hence, although there is class mixing as in the previous example, here one class dominates over the other. This is also reflected in the contour density plots, where the class *k* = 1 (magenta) resemble a near cube shape, with one edge much more diffused than other edges. The class *k* = 2 (cyan) on the other hand is very scattered in all facets. In Fig. 4(f), the color coding exhibits a clear correlation between outlying diameters and higher aspect ratios. A statistical summary is given in Table 3. This result provides a further demonstration of the flexibility of the soft classification scheme. Statistically identifying the proportions of nanocubes and quasi-nanocubes representative of the sample will enable accurate correlation between structure and properties, which is essential in the development of nanoparticles for magnetic and optical applications where shape monodispersity is crucial.

**Table 3:** Summary for Fe/Fe_2_O_3_ nanocubes shape analysis, where *k* denotes the shape class, ∑ and fraction are the effective number (total responsibility) and fraction of particles, diam. and AR are the particle diameter and aspect ratio

*k*	∑	Fraction	Diam. (nm)	AR
1	89.3	81%	10.7 ± 1.1	1.11 ± 0.05
2	20.3	19%	10.9 ± 1.9	1.27 ± 0.09

### 3.4 Separating aggregated nanoparticles – the binary DoG

As described earlier, it is not always possible or desirable to achieve well-dispersed nanoparticles and very often there is at least some fraction of particles that are touching, connected or aggregated. In fact, analogous problems persist in particle analysis in other fields.^58,59^ To further expand the applicability of our method to include aggregated particles, here we demonstrate a new method for analyzing aggregated particles which utilizes the contrast contained in the original TEM images (recall that most methods use only the binary image, including the methods of the present work up to this section). Our new method, which we coin the “binary DoG” for reasons that will become apparent, entails one simplifying assumption, namely, that the aggregates are composed of particles whose possible shapes are known up to an affine geometric transformation. In the example presented here, knowledge of the possible shapes comes from a prior analysis of the isolated particles. This is a reasonable assumption for many nanoparticle systems, since it should be unlikely that aggregated particles will have shapes entirely different from those of isolated particles.

**Fig. 5:**
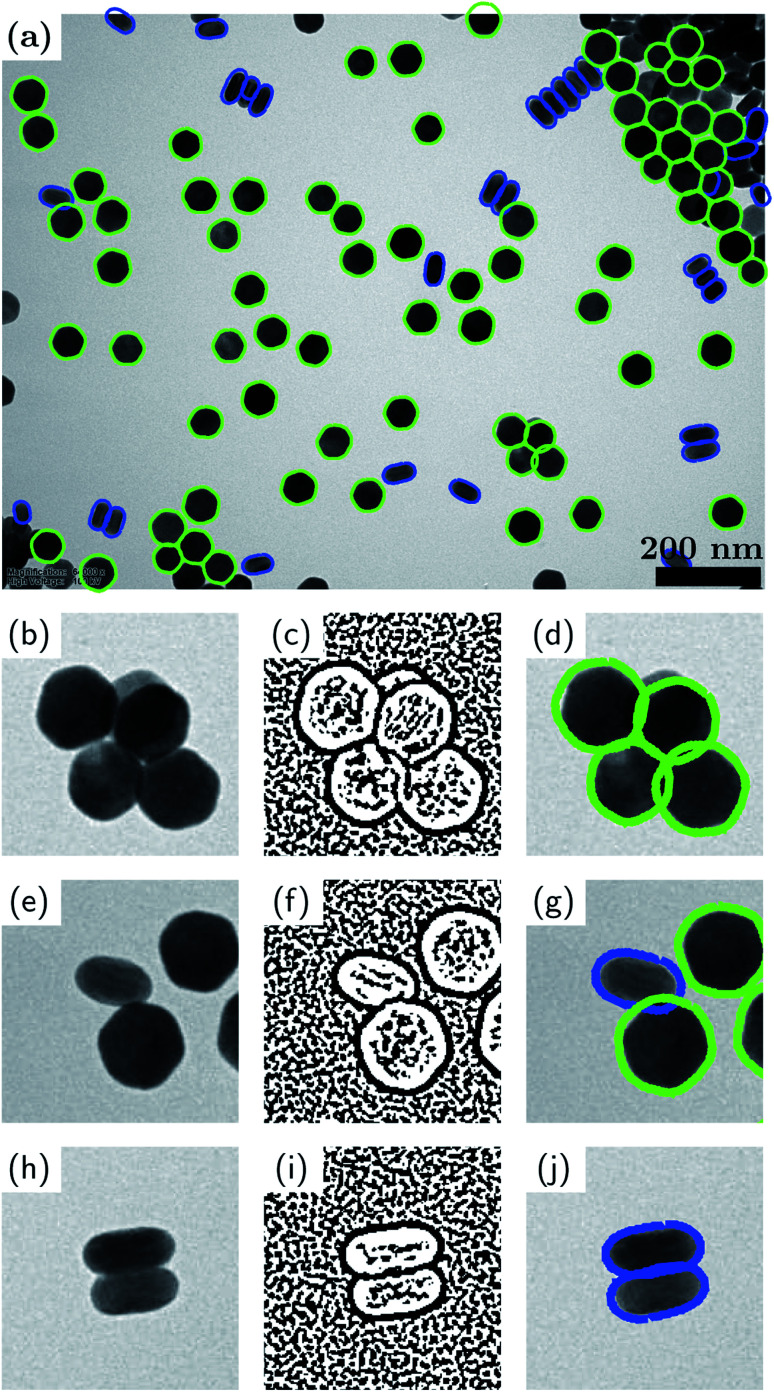
Binary-DoG method for shape analysis of aggregated particles. (a) Result of the method applied to the UCNPs considered in Section 3.1; (b, e and h) magnified views of touching, connected and aggregated particles; (c, f and i) corresponding binary DoGs; (d, g and j) corresponding contours.

Fig. 5 demonstrates our binary DoG method for the case of the UCNPs presented earlier in Section 3.1. Recall that this nanoparticle system consisted of two well-defined shapes, namely, hexagonal particles and rod-shaped particles. Fig. 5(a) shows the final result of the binary DoG method where the vast majority of particles are identified, now including aggregated particles.

The binary DoG method works by recognizing that the contrast in TEM images (BF-TEM or ADF-STEM) at nanometer resolution is often dominated by thickness contrast. Hence, the edges of the nanoparticles typically comprise regions where the Laplacian (loosely speaking the curvature) of the intensity has definite sign. These regions can be identified by first applying a difference-of-Gaussians (DoG) operator (which approximates the Laplacian operator), and then applying a binary operation to the resulting image based on its sign. The result of these two operations is a binary DoG image which represents the sign of the intensity curvature, and it can be very effective in separating overlapped/aggregated particles, as shown for the touching, connected and aggregated particles in Fig. 5(b, c), (e, f) and (h, i), respectively.

The last (but not least) step in extracting the shapes of the aggregated particles consists of fitting the possible contours to the binary DoG image. The possible contours are assumed to be known (in our example from an analysis of the isolated particles). The contours are fitted by optimizing their overlap with the binary DoG image. For example, in Fig. 5 an optimum overlap is achieved when a contour resides entirely within the black pixels of the binary DoG. During the fitting, the contours are allowed to undergo affine geometric transformations, which entail changes in nonisotropic scaling, skewness, orientation and position (each within certain limits). The greater flexibility afforded by affine transformations, as opposed to rigid-body transformations, allows for the fact that the particle shapes can appear slightly distorted in the binary DoG image. We find that the computation time of the fitting step can be greatly improved by utilizing a fast Fourier transform (FFT) algorithm along with the convolution theorem to simultaneously translate the contours and compute their overlap with the binary DoG. In the present example, there are two possible contours to be fitted: a hexagonal one and a rod-shaped one. Each of these contours was an average contour created from the contours of isolated particles that were classified previously (see Fig. 2). Fourier smoothing and resampling were applied to accomplish the contour averaging.

It is seen that the binary DoG method successfully separates the aggregated particles and correctly identifies the majority of particles in the image. The method overcomes many of the limitations of the previously reported methods such as “Erode and Flood”^59^ and “Convex Hull”,^60^ based on a reasonable assumption that the possible shapes of the aggregated particles are known.

## 4. Conclusions

We have demonstrated a new, quantitative and robust approach for the metrology of convex-shaped nanoparticles *via* soft classification machine learning of TEM images. Unlike existing approaches, our HuSC method, a probabilistic, soft classification method, can effectively and meaningfully classify a broad class of nanoparticle systems, from systems entailing distinct shapes, to systems entailing a continuum of non-distinct shapes. The use of (reduced) Hu moments also allows a simple and quantitative way to visualize the shape variations of convex nanoparticles. The HuSC method was demonstrated by means of three distinct scenarios covering a range of nanoparticle compositions, densities, shapes, and TEM imaging conditions. The method is capable of delivering a highly automated and highly accurate analysis of large numbers of nanoparticles. Moreover, with little modification, the method can be extended to accommodate non-convex-shaped particles, which will be presented in a future publication. In addition, we demonstrated a binary DoG method which is capable of analyzing the difficult case of aggregated nanoparticles, with a significantly higher success rate compared to previously reported methods. The ability to statistically analyze key nanoparticle performance parameters such as shape and size provides invaluable feedback for achieving synthesis control of nanoparticle properties. Even more importantly, such quantitative data enables deeper and more accurate understandings of shape-dependent properties and performance at the nanoscale. Finally, while here we have demonstrated our HuSC method for inorganic nanoparticles from TEM images, our method is generalizable to biological vesicles, like exosomes and liposomes, as well as particle analysis based on other types of microscopy, such as light microscopy.

## Author contributions

HW and CD developed and coded the image processing and machine learning algorithms; SLYC, HW and CD wrote the manuscript; SX, SC, JHC, SCL contributed the nanoparticle samples and carried out the TEM imaging.

## Conflicts of interest

There are no conflicts to declare.

## Supplementary Material

NA-003-D1NA00524C-s001
