# Supplementary material for: Metrology of convex-shaped nanoparticles via soft classification machine learning of TEM images
Source: Nanoscale Adv. 2021 Oct 13;3(24):6956–64. doi: 10.1039/d1na00524c (PMC9417281; doi:10.1039/d1na00524c)
Supplement: NA-003-D1NA00524C-s001 [file NA-003-D1NA00524C-s001.pdf]

Electronic Supplementary Material (ESI) for Nanoscale Advances.
This journal is © The Royal Society of Chemistry 2021

Supplementary Information for "Metrology of Convex-Shaped Nanoparticles via Soft Classification Machine Learning of TEM Images"

Haotian Wen
School of Materials Science and Engineering,
University of New South Wales, Sydney, NSW 2052, Australia

Xiaoxue Xu
School of Mathematical and Physical Sciences,
University of Technology, Sydney, Ultimo, NSW 2007, Australia

Soshan Cheong
Electron Microscope Unit, Mark Wainwright Analytical Centre,
University of New South Wales, Sydney, NSW 2052, Australia

Shen-Chuan Lo and Jung-Hsuan Chen
Material and Chemical Research Laboratories,
Industrial Technology Research Institute, Hsinchu, Taiwan

Shery L.Y. Chang
Electron Microscope Unit, Mark Wainwright Analytical Centre,
University of New South Wales, Sydney, NSW 2052, Australia and
School of Materials Science and Engineering,
University of New South Wales, Sydney, NSW 2052, Australia

Christian Dwyer
Electron Imaging and Spectroscopy Tools,
PO Box 506, Sans Souci, NSW 2219, Australia

S1. HU MOMENT SOFT CLASSIFICATION METHOD

In this section, we explain in detail the HuSC methodology. This methodology involves two main parts as shown in Fig. 1 in the main text. The first part is the image preprocessing that converts TEM images into particle contour datasets, which are then input to the classification process. The second part is the soft classification algorithm which classifies the nanoparticle contours and ultimately assigns a set of class "responsibilities" to each contour.

S1. Image preprocessing

S1. Background removal

The first step of pre-processing is to separate the particles from the background in the image. The raw image is firstly filtered and smoothed using Fourier filtering method to reduce the image noise. In TEM images, variations of the background can arise from variations in the thickness of the TEM support film, or from non-uniform electron microscope illumination. In order to obtain reliable background-removed images, we adopt a refined approach of dynamic thresholding [1]. The use of the dynamic thresholding is important in achieving accurate separation of the particles from the background. Rather than assuming a global (single value) background for the TEM image, this method measures the local background value within sub-regions of the image before applying the background removal.

S2. Binary image

The background-subtracted image is converted to a binary image by assigning a value 0 to all pixels with a value less than or equal to the background, while assigning a value of 1 to all pixels with a greater value. The resulting binary image forms an unambiguous representation of the locations of the nanoparticles for subsequent processing.

S3. Particle contour identification

Using the binary image, the particles' contours (outlines) are identified using the Canny edge detection algorithm [2]. The algorithm is applied to a Gaussian-smoothed version of the binary image. Edge detection is based on the local gradients within the image, with the edges located at the pixels with locally maximum values of the magnitude of the gradient.

S4. Filtering out overlapping particles

Filtering is applied to remove the contours of overlapping particles, as these contours do not represent the shapes of individual particles. Ensuring that all particle contours represent isolated particles (i.e., not overlapping or aggregated) is necessary for the correct operation of the classification algorithm. We use a straight-forward convexity filter method to filter out the aggregated/overlapping particles. We define the convexity of a contour as the ratio of the area enclosed by the contour and the area enclosed by the convex hull (closest convex approximation) of the contour. This approach assumes that the contours of isolated particles are convex (that is, that the particles themselves have a convex shape) but that contours of aggregated particles are non-convex. Hence by measuring and thresholding the convexity of the particle contours, aggregates can be detected [3], and then removed from the dataset. A typical threshold value is around 95%.

If the shapes of individual particles are non-convex, then it is possible to devise other simple filters which can work well. For example, since the area enclosed by the contour of a particle aggregate tends to be significantly larger than that of an isolated particle, thresholding of the area values can form an effective filter. A more sophisticated approach using hard classification of contour shapes is also possible.

In Section 3.4 of the main text, we show that the requirement of isolated particles can potentially be lifted by using a "binary DoG" method in which the contours of overlapped particles can be correctly identified. The binary DoG method is not limited to convex shapes.

S2. Soft classification unsupervised machine learning method

S1. Hu moments as shape descriptors

For a grey-scale image (or 2D probability density function), $\rho(x, y)$, the $(p+q)$th order image moments are defined as

$$m_{p,q} = \int_{-\infty}^{\infty}\int_{-\infty}^{\infty} x^p y^q \rho(x, y) dx dy. \tag{S1}$$

In the context of closed contours representing particle shapes, we can assume that $\rho(x, y)$ is binary valued so that the integral is limited to the area A enclosed by the contour

$$m_{p,q} = \iint_A x^p y^q dx dy. \tag{S2}$$

Given a contour specified as an order list of coordinates, Green's theorem in the plane greatly aids the calculation of the above integral.

The Hu moments $H_{p,q}$ are, by design, invariant with respect to translation, scaling and rotation [4]. To obtain these, we first define centered moments $m'_{p,q}$, which are the moments of the contour translated such that its center of mass $(m_{1,0}, m_{0,1}) = (0, 0)$ (this achieves translation invariance). Using the centered moments, we define normalized moments

$$\mu_{p,q} \equiv \frac{m'_{p,q}}{m_{0,0}\sqrt{m_{0,0}^{p+q}}}, \tag{S3}$$

(this achieves scaling invariance). Finally, using the normalized moments, the seven Hu moment invariants are given by

$$\begin{aligned}
H_1 &= \mu_{2,0} + \mu_{0,2} \\
H_2 &= (\mu_{2,0} - \mu_{0,2})^2 + 4\mu_{1,1}^2 \\
H_3 &= (\mu_{3,0} - 3\mu_{1,2})^2 + (3\mu_{2,1} - \mu_{0,3})^2 \\
H_4 &= (\mu_{3,0} + \mu_{1,2})^2 + (\mu_{2,1} + \mu_{0,3})^2 \\
H_5 &= (\mu_{3,0} - 3\mu_{1,2})(\mu_{3,0} + \mu_{1,2})((\mu_{3,0} + \mu_{1,2})^2 - 3(\mu_{2,1} + \mu_{0,3})^2) \\
&\quad + (3\mu_{2,1} - \mu_{0,3})(\mu_{2,1} + \mu_{0,3})(3(\mu_{3,0} + \mu_{1,2})^2 - (\mu_{2,1} + \mu_{0,3})^2) \\
H_6 &= (\mu_{2,0} - \mu_{0,2})((\mu_{3,0} + \mu_{1,2})^2 - (\mu_{2,1} + \mu_{0,3})^2) + 4\mu_{1,1}(\mu_{3,0} + \mu_{1,2})(\mu_{2,1} + \mu_{0,3}) \\
H_7 &= (3\mu_{2,1} - \mu_{0,3})(\mu_{3,0} + \mu_{1,2})((\mu_{3,0} + \mu_{1,2})^2 - 3(\mu_{2,1} + \mu_{0,3})^2) \\
&\quad - (\mu_{3,0} - 3\mu_{1,2})(\mu_{2,1} + \mu_{0,3})(3(\mu_{3,0} + \mu_{1,2})^2 - (\mu_{2,1} + \mu_{0,3})^2)
\end{aligned} \tag{S4}$$

The first two Hu moments have the simple interpretation given in the main text. Generally, we find that discrimination of more complex shapes requires the use of higher order Hu moments.

There also exist a range of other well-established shape descriptors which are compatible with soft classification, as cited in the main text.

S2. Gaussian mixture model soft classification method

Given a set of N particle contours, we can represent each one of them by its (possibly reduced subset of) Hu moments, as described above, so that the set of N contours is represented by a set of points $\mathbf{x}_1, \ldots, \mathbf{x}_N$ in the multidimensional "Hu space." This concept is quite general in that it applies equally well to other shape descriptors, so that we may refer

to the set of contours as represented by a set of points in a more general multidimensional "shape space."

We assume that the set of (data) points $\mathbf{x}_1, ..., \mathbf{x}_N$ represents some underlying probability distribution $p(\mathbf{x})$ in the shape space. Following the treatment outlined in Ref. [5], soft classification using a Gaussian mixture model consists of finding the most likely superposition of K Gaussian densities that best reproduces the underlying probability distribution $p(\mathbf{x})$. The superposition of Gaussians is given by

$$p(\mathbf{x}) = \sum_{k=1}^{K} \pi_k \mathcal{N}(\mathbf{x}|\boldsymbol{\mu}_k, \boldsymbol{\Sigma}_k), \tag{S5}$$

where each Gaussian density $\mathcal{N}$ has its own mean $\boldsymbol{\mu}_k$, covariance $\boldsymbol{\Sigma}_k$, and mixing coefficient (weighting) π_k. For a given set of points data points $\mathbf{x}_1, ..., \mathbf{x}_N$, optimization of the Gaussian mixture model, that is, optimization of the quantities $\boldsymbol{\pi} = \pi_1, \ldots, \pi_K$, $\boldsymbol{\mu} = \boldsymbol{\mu}_1, \ldots, \boldsymbol{\mu}_K$, and $\boldsymbol{\Sigma} = \boldsymbol{\Sigma}_1, \ldots, \boldsymbol{\Sigma}_K$, can be achieved by a maximum likelihood solution, where the log-likelihood function is given as

$$\ln p(\mathbf{x}_1, ..., \mathbf{x}_N|\boldsymbol{\pi}, \boldsymbol{\mu}, \boldsymbol{\Sigma}) = \sum_{n=1}^{N} \ln \left\{ \sum_{k=1}^{K} \pi_k \mathcal{N}(\mathbf{x}_\mathbf{n}|\mu_k, \Sigma_k) \right\}. \tag{S6}$$

The essential goal of the above procedure is that it enables a soft classification of each data point (contour) $\mathbf{x}_n$ in terms of a set of K responsibilities $p(k|\mathbf{x}_n)$, defined as

$$p(k|\mathbf{x}_n) = \frac{\pi_k \mathcal{N}(\mathbf{x}_n|\boldsymbol{\mu}_k, \boldsymbol{\Sigma}_k)}{\sum_{k'=1}^{K} \pi_{k'} \mathcal{N}(\mathbf{x}_n|\boldsymbol{\mu}_{k'}, \boldsymbol{\Sigma}_{k'})}. \tag{S7}$$

The responsibility $p(k|\mathbf{x}_n)$ is the probability of the unobserved mixture component k given the observed data point $\mathbf{x}_n$. Being probabilities, the responsibilities satisfy $0 \leq p(k|\mathbf{x}_n) \leq 1$ and $\sum_{k=1}^{K} p(k|\mathbf{x}_n) = 1$ (which is easily seen from their definition above). In the three examples given in the main text, we use $K = 2$ to accomplish the soft classification of particle contours in Hu space.

S2. ANALYSIS USING MULTIPLE TEM IMAGES

Here we present an extended example of the HuSC metrology analysis that was presented in Section 3.1 of the main text. This extension is simply intended to demonstrate that the method produces consistent results when applied to multiple TEM images, as would be expected, and as would be necessary in an application of the method to perform a detailed analysis of a particular nanoparticle system. Here we show the results obtained from an

k	$\sum$	Fraction	Diam. (nm)	AR
1	91	85.0%	64.4±3.3	1.03±0.02
2	16	15%	49.1±5.3	1.68±0.2

TABLE S1. Summary for UCNPs shape analysis, where k denotes the shape class, $\sum$ and Fraction are the effective number (total responsibility) and fraction of particles, Diam. and AR are the particle diameter and aspect ratio.

additional 4 TEM images of the UCNP system, that contain 110 additional nanoparticles (in comparison to 43 nanoparticles shown in Fig. 2). The 4 TEM images of the UCNP sample, shown in Fig. S1(a), were acquired using exactly the same batch of nanoparticles. As in Section 3.1, the HuSC method was applied assuming two shape classes. Once again, we see that in this case, where the particles have distinct, well-defined shapes, the soft classification reduces to an essentially hard classification of particles shapes: hexagonal (magenta) and rod-shaped (cyan). Detailed shape and size analysis, given as the particle shape eigenvalue scatter plot in Fig. S1(d), and the size histogram in Fig. S1(e), respectively, show essentially the same distribution as given in Fig. 2, as expected. Again, the particles in this example fall very close to the ellipse line (dashed line in Fig. S1(d)), with class $k = 1$ (magenta) focused around aspect ratios in the range 1.0–1.1, and class $k = 2$ spread between 1.5 to 2.0 (except for one particle). The size distribution of $k = 1$ is around 58-68 nm, and that for $k = 2$ around 46-52 nm. These distributions are similar to those presented in Fig.2, with a minor difference of a single particle in class $k = 2$ that is much smaller ($\approx$ 32 nm) and has a smaller aspect ratio of $\approx$ 1.25 (seen near the center of lower-left TEM image). A summary of the statistics is given in Table S1, to be compared with Table 1 in the main text.

[1] E. Davies *et al.*, Algorithms, Practicalities **2** (1990).
[2] J. Canny, IEEE Transactions on Pattern Analysis and Machine Intelligence , 679 (1986).
[3] C. Park, J. Z. Huang, J. X. Ji, and Y. Ding, IEEE transactions on pattern analysis and machine intelligence **35**, 1 (2012).
[4] M. K. Hu, IRE Trans. Info. Theory **8**, 179 (1962).
[5] C. Bishop, *Pattern Recognition and Machine Learning* (Springer MIHE, 2006).

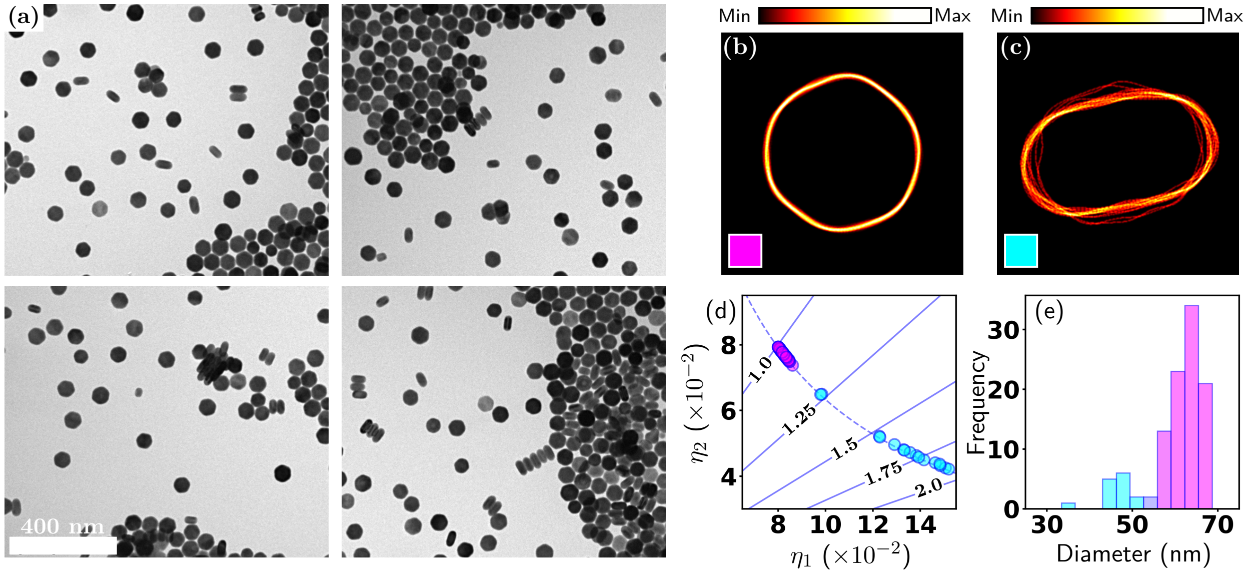

FIG. S1. Demonstration of the HuSC method using multiple TEM images. The sample is the same UCNP sample as in Section 3.1 of the main text. (a) Four BF-TEM images; (b,c) density plots of scale and orientation matched contours; (d) scatter plot of eigenvalues; (e) distribution of effective diameters. Color coding reflects the results of the shape classification.
